# N-rich chitosan-derived porous carbon materials for efficient CO_2_ adsorption and gas separation

**DOI:** 10.3389/fchem.2023.1333475

**Published:** 2023-12-14

**Authors:** Han Min, Ke Zhang, Zhongya Guo, Fengyao Chi, Lili Fu, Bin Li, Xueyi Qiao, Shuang Wang, Shaokui Cao, Bing Wang, Qingxiang Ma

**Affiliations:** ^1^ Henan Key Laboratory of Advanced Nylon Materials and Application, School of Materials Science and Engineering, Zhengzhou University, Zhengzhou, China; ^2^ Zhengzhou Tobacco Research Institute of CNTC, Zhengzhou, China; ^3^ State Key Laboratory of High-efficiency Utilization of Coal and Green Chemical Engineering, Ningxia University, Yinchuan, China

**Keywords:** chitosan, nitrogen-doped porous carbon, CO_2_ adsorption, gas adsorption and separation, biomass

## Abstract

Capturing and separating carbon dioxide, particularly using porous carbon adsorption separation technology, has received considerable research attention due to its advantages such as low cost and ease of regeneration. In this study, we successfully developed a one-step carbonization activation method using freeze-thaw pre-mix treatment to prepare high-nitrogen-content microporous nitrogen-doped carbon materials. These materials hold promise for capturing and separating CO_2_ from complex gas mixtures, such as biogas. The nitrogen content of the prepared carbon adsorbents reaches as high as 13.08 wt%, and they exhibit excellent CO_2_ adsorption performance under standard conditions (1 bar, 273 K/298 K), achieving 6.97 mmol/g and 3.77 mmol/g, respectively. Furthermore, according to Ideal Adsorption Solution Theory (IAST) analysis, these materials demonstrate material selectivity for CO_2_/CH_4_ (10 v:90 v) and CO_2_/CH_4_ (50 v:50 v) of 33.3 and 21.8, respectively, at 1 bar and 298 K. This study provides a promising CO_2_ adsorption and separation adsorbent that can be used in the efficient purification process for carbon dioxide, potentially reducing greenhouse gas emissions in industrial and energy production, thus offering robust support for addressing climate change and achieving more environmentally friendly energy production and carbon capture goals.

## Graphical Abstract

**Figure:**
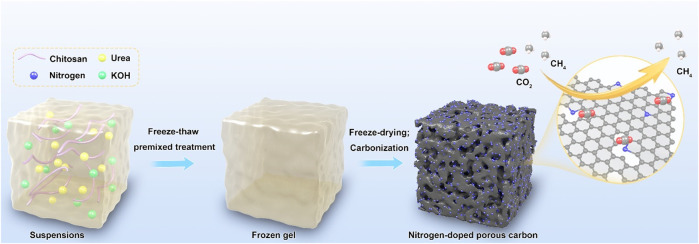
The nitrogen-doped porous carbon materials synthesized by the one-step carbonization activation method of freeze-thaw premixed treatment had extremely narrow pore size distribution and high nitrogen content, thus improving the adsorption and separation performance of the materials for CO_2_/CH_4_ gas mixtures.

## 1 Introduction

Over the past few decades, the extensive consumption of fossil fuels such as coal and oil has led to a significant increase in the concentration of carbon dioxide (CO_2_) in the atmosphere (Singh et al., 2019). These substantial CO_2_ emissions have adverse impacts on Earth’s climate, including accelerating sea-level rise and continuous glacier melting. With the rapid development of human society, the demand for energy continues to grow, while the reserves of fossil fuels like coal and oil are gradually depleting, further increasing the need for renewable alternative energy sources. Biogas was reliable renewable energy, generally produced by the anaerobic digestion of biomass. Biogas was mainly composed of CH_4_, CO_2_ and other components (Adnan et al., 2019). However, the existence of high concentration CO_2_ will reduce its calorific value and then hinder its practical application (Wang et al., 2015). To effectively utilize biogas and reduce air pollution, it was very urgent and vital to capture CO_2_ and recover CH_4_ from CO_2_/CH_4_ mixture gas. Carbon capture and CO_2_ separation technologies have thus become critical fields for reducing greenhouse gas emissions and achieving more environmentally sustainable energy production goals (Bernardo et al., 2021).

In this context, we place a particular emphasis on the capture and separation of CO_2_, especially through the use of porous carbon adsorption separation technology. Highly porous and nitrogen-doped porous carbon materials have demonstrated outstanding performance in CO_2_ adsorption and separation (Petrovic et al., 2021; Kielbasa, 2023; Li et al., 2023). Specifically, micropores smaller than 1 nm are crucial for enhancing CO_2_ adsorption and selectivity (Liu et al., 2020). Nitrogen-doped porous carbon materials are considered ideal candidates for CO_2_ capture and gas separation due to their enhanced interactions and the ability to selectively absorb acidic CO_2_ molecules (Yang et al., 2018; Wu et al., 2023).

However, current methods for preparing porous carbon materials often face two challenges when balancing high adsorption performance and high separation performance: first, they tend to produce materials with a wide pore size distribution, and second, they result in lower nitrogen content in the materials. These challenges make it difficult to obtain porous carbon materials that simultaneously exhibit high adsorption capacity and high selectivity. Therefore, the preparation of nitrogen-doped porous carbon adsorbents with high adsorption capacity and selectivity remains a challenging task.

In this study, we propose a one-step carbonization activation method using chitosan as a carbon source, urea as a nitrogen source, and potassium hydroxide as an activator with freeze-thaw pre-mix treatment to synthesize porous carbon materials with an extremely narrow pore size distribution (pore size <1 nm) and high nitrogen content (up to 13.08 wt%). Chitosan dissolves in alkaline conditions through freeze explosion, driven by its water absorption mechanism. After full water absorption, freezing at a low temperature alters the physical state, breaking weak hydrogen bonds between chitosan molecules. Addition of alkaline solvents like urea or ammonia disrupts hydrogen bonds further. Repeated freezing and thawing achieve dissolution, ensuring uniform distribution of nitrogen-doped agent and activator in the material. This enhances their adsorption and separation performance for gas mixtures. These porous carbon materials are expected to play a significant role in applications such as gas storage and gas purification, offering a potential solution for reducing greenhouse gas emissions and achieving more environmentally sustainable energy production goals.

## 2 Experiment section

### 2.1 Chemicals

Chitosan, urea, KOH and HCl were all analytically pure and purchased from China Pharmaceutical Group Co., Ltd. Deionized water was self-made in the laboratory.

### 2.2 Synthesis methods

9.6 g chitosan, 16 g urea, and KOH were dissolved in 80 mL of deionized water and stirred at room temperature for 30 min to obtain suspensions with the mass ratio of KOH/chitosan at 1 and 2, respectively. The suspensions were frozen at −34°C for 48 h, and the frozen solids were thawed at room temperature to obtain gelatinous substances. The solids obtained by direct freeze-drying were carbonized for 2 h at 600°C, 700°C, and 800°C, respectively. The heating rate was controlled at 10°C/min. After KOH was removed by dilute hydrochloric acid, the material was washed with deionized water to neutral and dried at 105°C for 12 h. The resulting material was denoted as FDCK-x-t (x was the KOH/chitosan mass ratio, t was the activation temperature).

9.6 g chitosan was put into a tubular furnace and carbonized at 700°C for 2 h. 9.6 g chitosan, 9.6 g KOH, and 16 g urea were ground in a mortar to a uniformly mixed powder, which was carbonized at 700°C for 2 h. The heating rate was controlled at 10°C/min. After washing with dilute hydrochloric acid and deionized water, it is dried in an oven at 105°C for 12 h. The obtained carbon materials were recorded as C-700 and CK-1-700 as the control group.

### 2.3 Characterization

Scanning electron microscope (SEM, Hitachi SU8010, Japan) and transmission electron microscopy (TEM, JEM-2100F, Japan) were used to characterize the microstructure and morphology of the samples. The material’s internal structure was further characterized by X-ray diffraction (XRD, SmartLab, Japan). The correlation analysis of C, N, and O elements in the materials was carried out by elemental analyzer (Elementar-UNICUBE, Germany) and X-ray photoelectron spectroscopy (XPS, Thermofisher Nexsa, America).

The Micromeritics ASAP 2460 adsorption apparatus measured the samples’ N_2_ adsorption-desorption isotherms (77 K). The Brunauer Emmett Teller (BET) method was used to calculate the specific surface area of the sample. The nonlocal density functional theory (NLDFT) method derived pore size distribution (PSD) from the N_2_ isotherm adsorption branch, assuming a slit pore model. Under the relative pressure of 0.99, the total pore volume of the sample was calculated by N_2_ adsorption capacity, and the area and volume of micropores were estimated by the t-plot method. The pore volume of narrow micropores (<1 nm) was obtained from the CO_2_ adsorption data at 273 K.

### 2.4 Related calculation

#### 2.4.1 IAST

The ideal adsorption solution theory proposed by Myers and Prausnitz was one of the mainstream models widely used to evaluate the adsorption selectivity of binary gas mixtures in recent years (Li J. et al., 2022; Yun et al., 2022). The adsorption selectivity of binary mixture gas can be defined as:

$$s_{1/2}=\left(q_{1}/q_{2}\right)/\left(p_{1}/p_{2}\right)$$ (1)

where q_1_ and q_2_ were the adsorption amounts of component 1 and component 2 in the binary gas mixture under partial pressures p_1_ and p_2,_ respectively.

#### 2.4.2 The isosteric heat of adsorption (*Q*_*st*_)

The heat of adsorption referred to the heat released when the temperature was certain. The adsorbate was adsorbed to the adsorbent. It reflected the energy variation, the heterogeneity of the material surface, and the interaction between adsorbent and gas molecules during the adsorption process, which was an important index to evaluate the regeneration performance of adsorbent materials. Isothermal adsorption heat was difficult to obtain by direct measurement and was generally accepted by the Clausius-Clapeyron equation (Qin et al., 2019).

$$Q_{st}=\frac{RT_{1}T_{2}\ln\left(P_{1}/P_{2}\right)}{T_{1}-T_{2}}$$ (2)

where T_i_ was the adsorption temperature; P_i_ was the pressure corresponding to T_i_ when the same adsorption capacity was reached; R was the ideal gas constant.

## 3 Results and discussion

### 3.1 Analysis of morphology, phase structure and surface chemical properties

**FIGURE 1:**
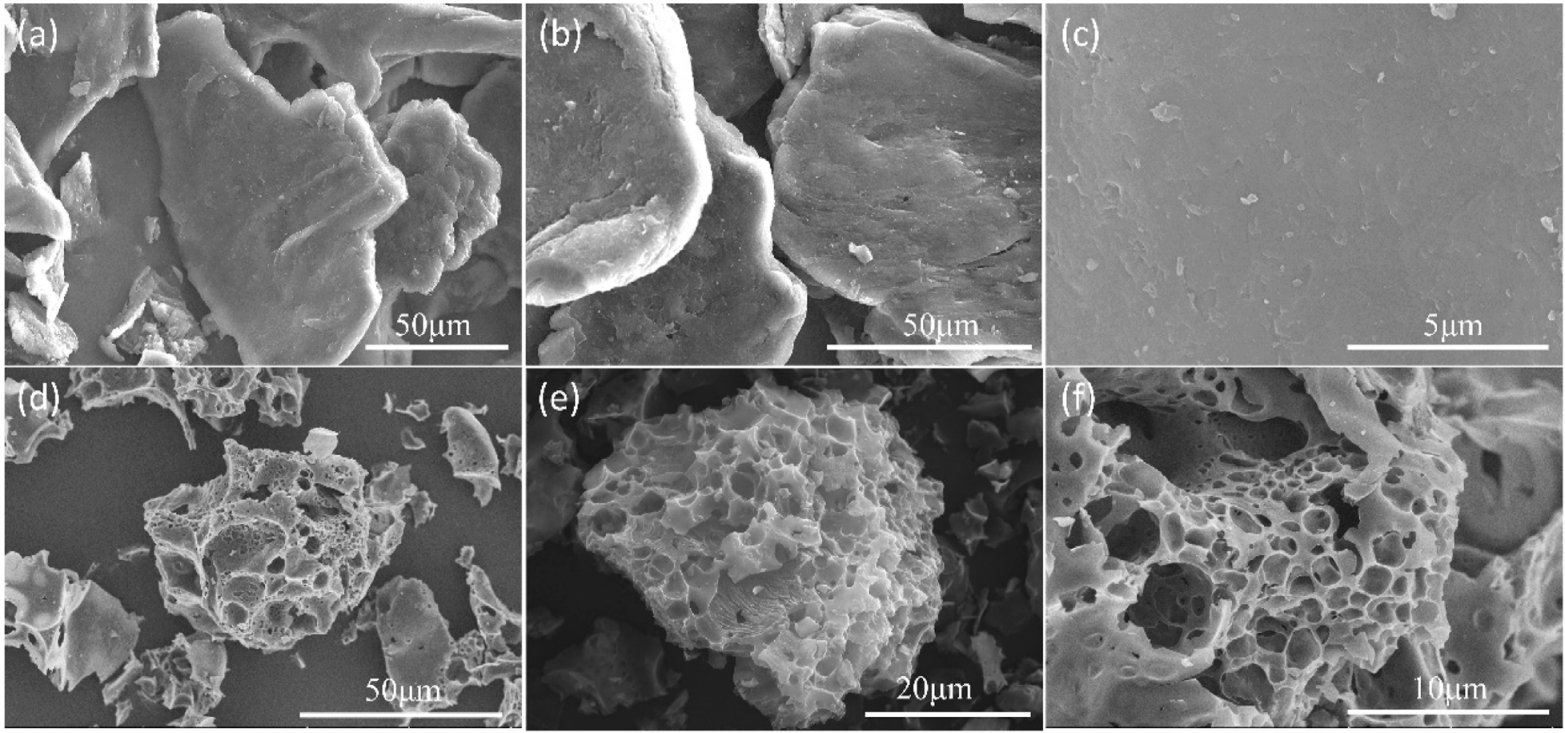
SEM images of C-700 **(A–C)**, and FDCK-1-700 **(D–F)**.

**FIGURE 2:**
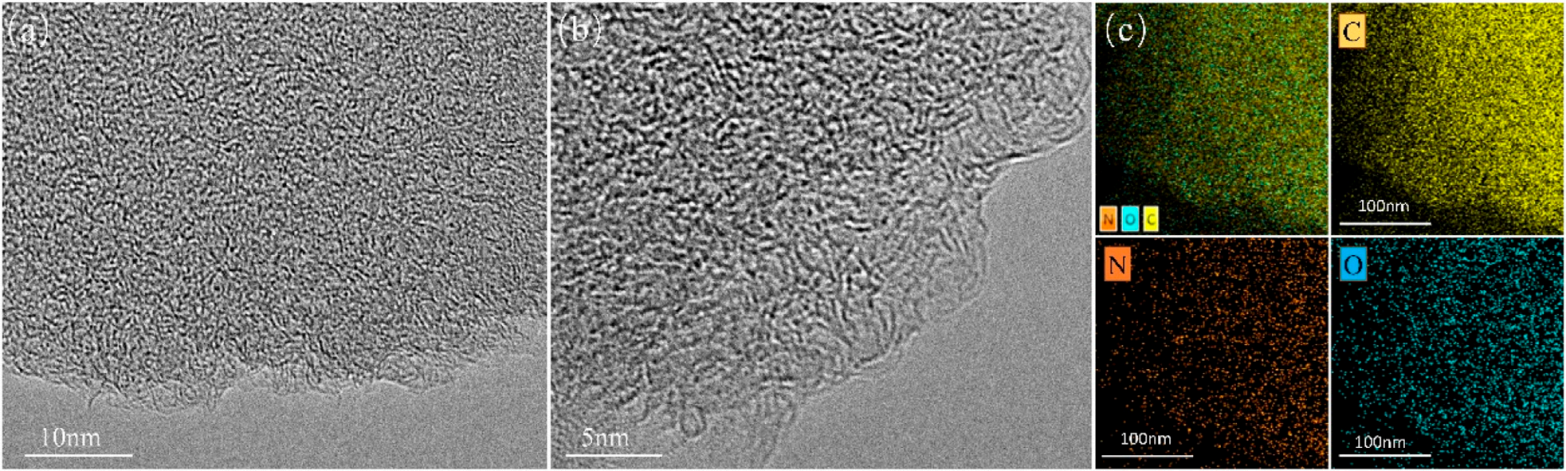
TEM images **(A,B)**, and C/N/O elemental mapping **(C)** of FDCK-1-700 .

SEM was used to observe morphologies of C-700 and representative nitrogen-doped porous carbon FDCK-1-700. As shown in Figure 1, C-700 had a smooth, flat surface. FDCK-1-700 modified by urea and KOH showed a prominent honeycomb structure. Microscopic morphologies of FDCK-1-700 were further characterized by TEM (Figure 2). FDCK-1-700 had abundant wormlike micropores with disordered distribution, indicating the amorphous structure of the sample. In addition, according to the EDS element mapping diagram (Figure 2C), the uniform distributions of N and O elements in FDCK-1-700 were confirmed.

XRD was used to study the crystal structure of FDCK-1-700, as shown in Supplementary Figure S1A. Wide and weak peaks were observed at 24.5° and 43.5°, respectively, corresponding to (002) and (100) planes of amorphous graphite carbon (Qin et al., 2019). The results showed that the carbon material was amorphous, consistent with TEM observation.

The doped nitrogen of the material was evaluated by elemental analysis. The results of the element analysis are shown in Table 1. By comparing the nitrogen content of FDCK-1-t, the nitrogen content of FDCK-1-600 was the highest, being 13.08 wt%. It was noteworthy that compared with CK-1-700 (3.97 wt%), FCDK-1-700 showed a higher nitrogen content (7.07 wt%), demonstrating the superiority of nitrogen content for the one-step carbonization activation method of freeze-thaw premixed treatment. Under the same KOH/chitosan mass ratio, carbon material’s nitrogen content decreasedwith the activation temperature increase mainly because the destroyed N-containing functional groups increased with temperature (Rao et al., 2019). For example, the nitrogen content decreased from 13.08 wt% on FDCK-1-600 to 4.45 wt% on FDCK-1-800. Nitrogen content decreased from 6.47 wt% on FDCK-2-600 to 0.38 wt% on FDCK-2-800. The shallow nitrogen content of FDCK-2-800 may be caused by the high proportion of activators and the increase in activation temperature.

**TABLE 1:** Elemental analysis.

Specimens	N (wt%)	C (wt%)	O (wt%)
C-700	10.31	79.35	7.86
CK-1-700	3.97	79.99	16.94
FDCK-1-600	13.08	65.31	17.58
FDCK-1-700	7.71	76.89	11.95
FDCK-1-800	4.45	87.11	6.10
FDCK-2-600	6.47	67.75	21.76
FDCK-2-700	1.21	82.63	11.41
FDCK-2-800	0.38	87.24	5.10

**FIGURE 3:**
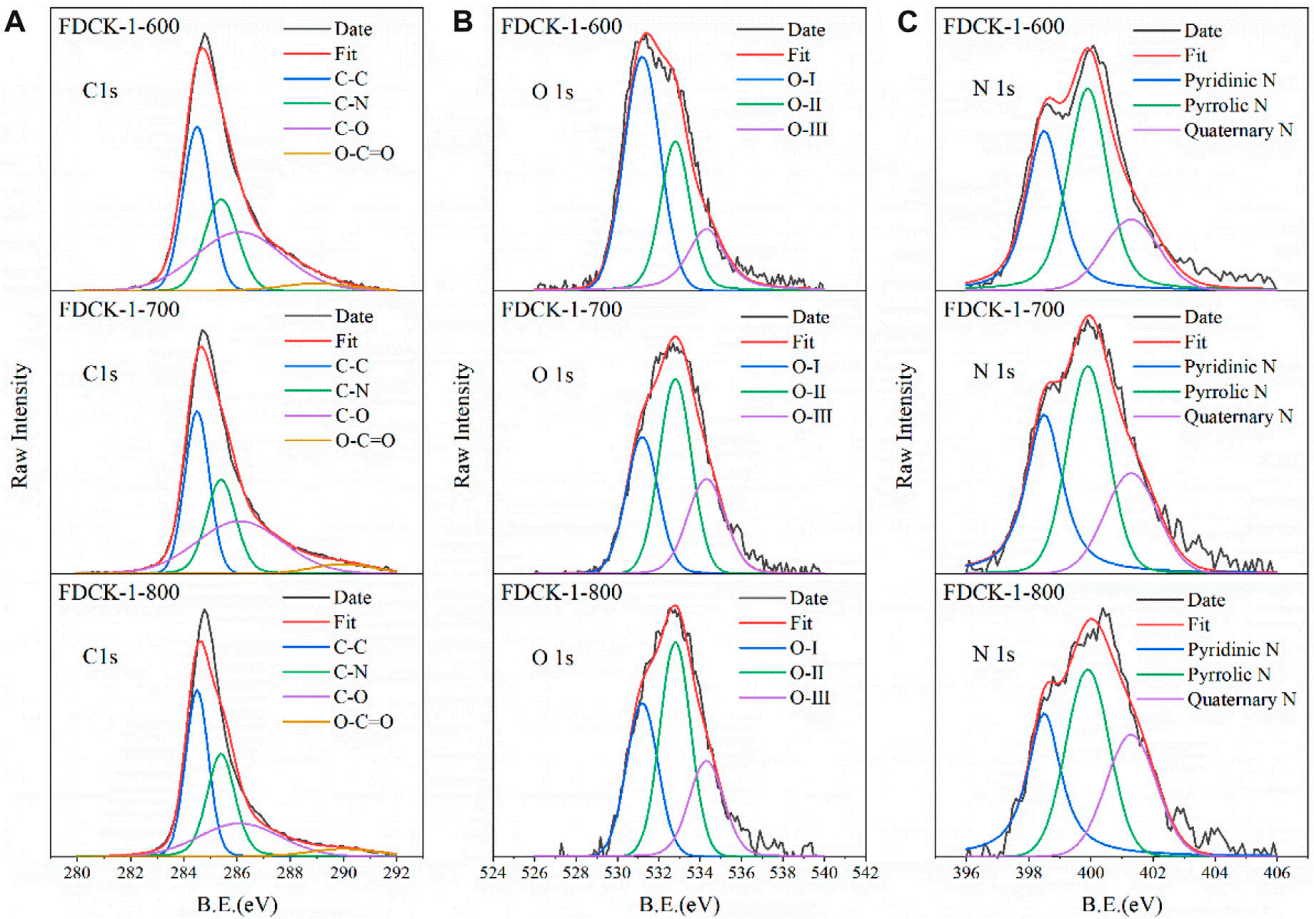
C 1s XPS patterns **(A)**, N 1s XPS patterns **(B)**, O 1s XPS patterns **(C)**.

XPS further analyzed the surface chemical properties of the materials. Supplementary Figure S1B was the XPS patterns of FDCK-x-t, we can observe three kinds of different peaks: C1s peaks at 283.5 eV, O1s peaks at 531.2 eV, and an N1s peaks at 398.8 eV. Figure 3 was the peak fitting results of the high-resolution C1s, N1s, and O1s atlas. FDCK-x-t high-resolution C1s spectrum (Figure 3A) could be fitted into four characteristic peaks at 285.2 eV, 286.2 eV, 288.0 eV, and 290.1 eV, corresponding to C-O, C-O, C-C, and O-C=O, respectively (Cai et al., 2018). FDCK-x-t high-resolution O1s spectrum (Figure 3B) could be fitted into three characteristic peaks at 530.0 eV, 532.6 eV, and 534.3 eV, corresponding to C-O, C-OH, and carboxyl groups, respectively (Rehman and Park, 2019). Oxygen-containing functional groups (especially C-OH) could increase the electron density on the surface of carbon materials and make more CO_2_ molecules enter the porous carbon materials through electrostatic adsorption, which further improved the CO_2_ adsorption performance of materials.

FDCK-x-t high-resolution N 1s spectrum (Figure 3C) could be fitted into three characteristic peaks at 398.4 eV, 399.9 eV, and 401.1 eV, corresponding to pyridinic-N, pyrrolic-N, and quaternary-N, respectively (Gao et al., 2016). Pyridinic-N and pyrrolic-N were components of six-membered and five-membered ring systems, respectively. Pyridinic-N could transfer one electron, and pyrrolic-N could provide two electrons to the π-system (Pels et al., 1995). Thus, these nitrogen functional groups exhibited the basic properties of lewis bases and played a role in captured lewis-acidic CO_2_ molecules. Supplementary Table S1 showed the relative nitrogen content of the porous carbon materials. It could be seen that the relative content of quaternary-N in the adsorbent increased gradually with the increase of activation temperature. This may be due to the rising temperature, which converted part of pyridinic-N and pyrrolic-N into the more stable quaternary-N.

### 3.2 Pore structure

**FIGURE 4:**
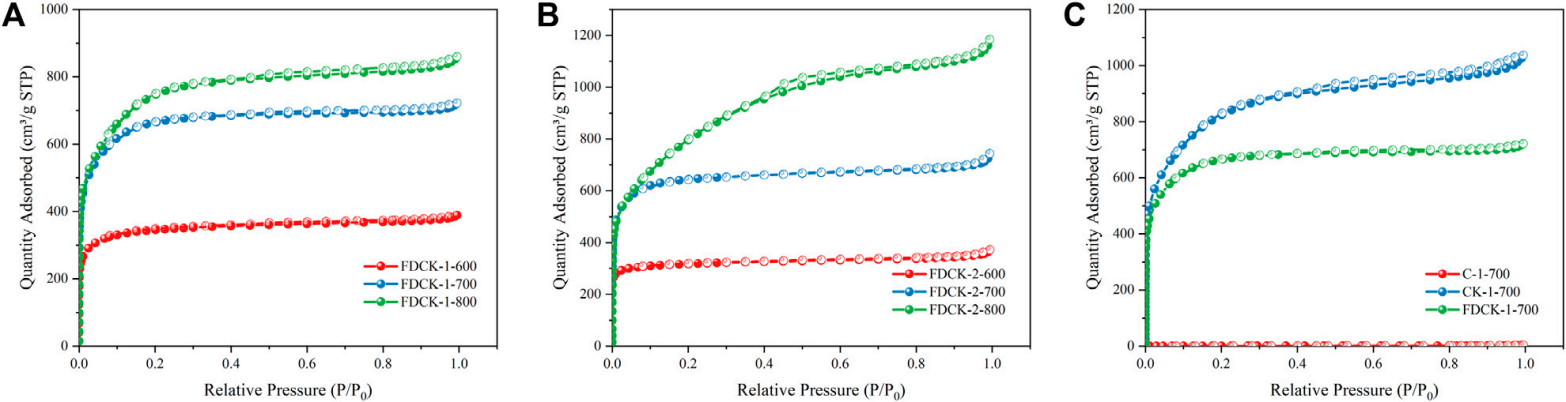
N_2_ adsorption-desorption isotherms at 77 K, FDCK-1-t **(A)**, FDCK-2-t **(B)**, and Contrast curve of different materials **(C)**.

**FIGURE 5:**
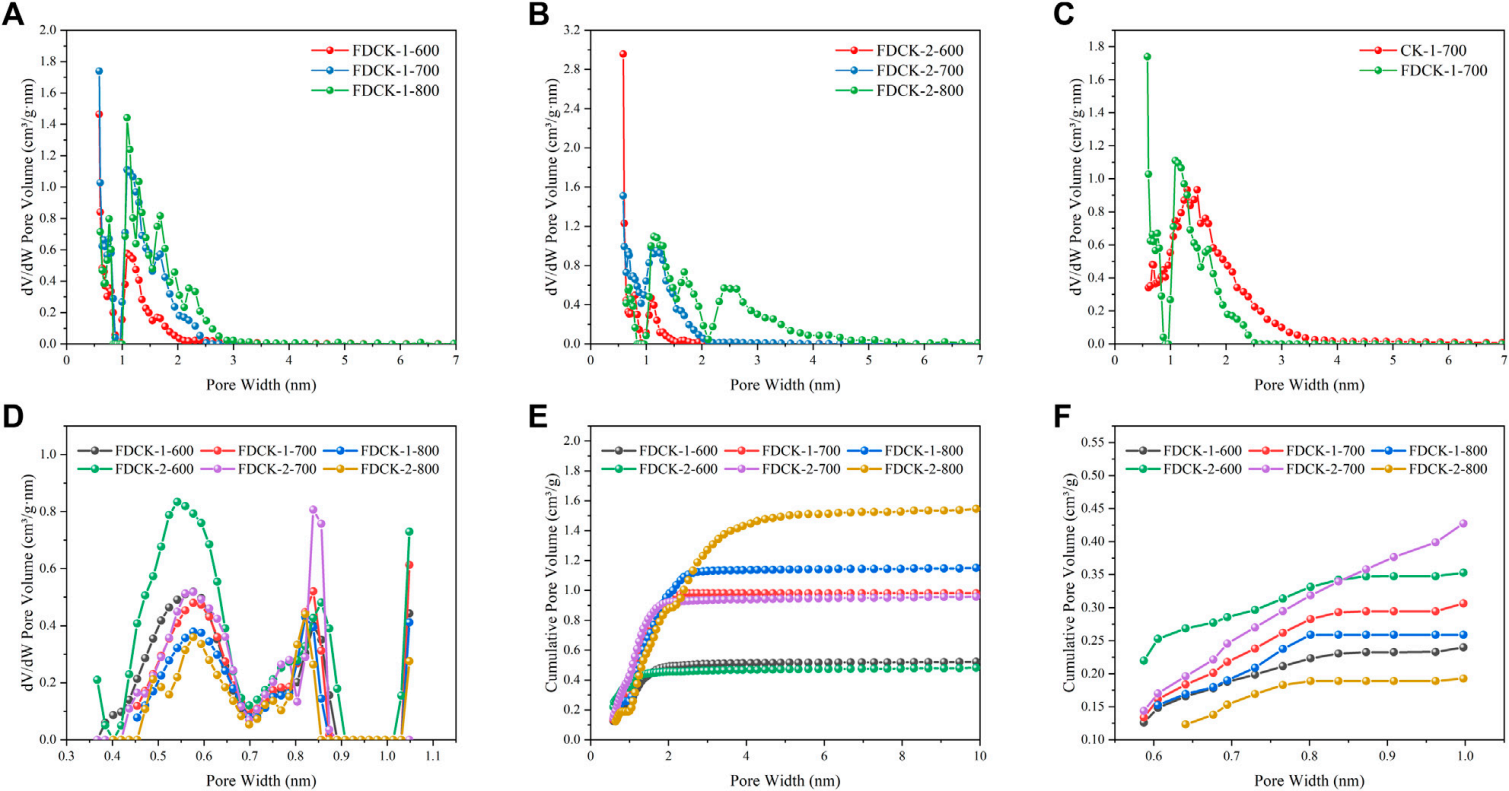
NLDFT pore size distribution curves **(A–C)**, the extremely narrow micropore distribution curves **(D)**, cumulative pore volume curves **(E, F)**.

The pore structure of the adsorbent was studied by an N_2_ adsorption-desorption isotherm at 77 K. As shown in Figure 4, FDCK-x-t showed sharp N_2_ absorption at low pressure, and then sharp “knees” appeared at P/P_0_ <0.2, followed by the formation of the adsorption platform. According to IUPAC classification, it belonged to the I type isotherm, indicating that the pore structure of FDCK-x-t was mainly microporous. As shown in Figure 4C, no obvious N_2_ absorption was observed on the N_2_ adsorption-desorption isotherm for C-700, indicating that the porosity of chitosan carbon was very low without being modified by urea and KOH. CK-1-700 showed rapid N_2_ absorption at low pressure, followed by slow N_2_ absorption and a certain hysteresis loop, indicating that it had a certain amount of mesopores. FDCK-1-700 showed a sharp N_2_ absorption under low pressure, followed by a sharp “knee” and an adsorption platform, and the adsorption curve and desorption curve basically coincide. Pore size distribution and cumulative pore volume of FDCK-x-t in Figure 5 also confirmed that they were mainly microporous, and there were a large number of extremely narrow micropores (pore size <1 nm) distribution. It was worth noting that FDCK-1-700 prepared by a one-step carbonization activation method of freeze-thaw premixed treatment had a high distribution of extremely narrow micropores, while CK-1-700 prepared by physical mixing under the same conditions had a more significant proportion of micropores (1 nm∼2 nm) and a certain amount of mesoporous pores (2 nm∼3 nm) (Figure 5C). The results indicated that the superiority of a one-step carbonization activation method of freeze-thaw premixed treatment for customizing extremely narrow microporous nitrogen-doped carbon materials. It could be seen that micropores of FDCK-x-t smaller than 1 nm were mainly concentrated in 0.5∼0.6 nm and 0.8∼0.9 nm (Figure 5D).

The specific surface area and pore volume parameters of C-700, CK-700, and FDCK-x-t are shown in Table 2. The specific surface area and total pore volume of the FDCK-x-t up to 2,897 m^2^/g and 1.83 cm^3^/g. The micropore area and micropore volume of FDCK-x-t reached the maximum at 700°C and decreased when the activation temperature increased to 800°C. This may be caused by the collapse of microporous channels due to high activation temperature. The results showed that 700°C was more suitable for carbonization activation of carbon precursors, which was consistent with previously reported results.

**TABLE 2:** Structural parametres of adsorbents.

Specimens	^a^S_BET_ (m^2^/g)	^b^S_mic_ (m^2^/g)	Pore volume (cm^3^/g)	
			^c^V_Total_	^b^V_< 2 nm_
C-700	2.94		—	—
CK-700	3,038	2,468	1.60	1.07
FDCK-1-600	1,318	1,155	0.60	0.46
FDCK-1-700	2,457	2,159	1.12	0.90
FDCK-1-800	2,680	2,001	1.33	0.87
FDCK-2-600	1,255	1,134	0.58	0.44
FDCK-2-700	2,464	2,224	1.15	0.89
FDCK-2-800	2,897	592	1.83	0.22

^a^ Specific surface area calculated by the BET method.

^b^ Micropore volume calculated by the t-plot method.

^c^ Total pore volume calculated at P/P_0_ = 0.99.

Chitosan could be dissolved in alkaline conditions by freeze explosion according to its water absorption mechanism. After chitosan fully absorbs water, the free water contained in it was frozen at a low temperature. Through the change in the physical state of water, the weak hydrogen bonds between chitosan molecules were dissociated. A small molecule of urea and hydrated metal ions could occupy the sites in chitosan molecules to form hydrogen bonds when alkaline solvents such as urea and KOH were added. Repeated freezing and thawing destroy the hydrogen bonds between chitosan molecules to achieve dissolution (Fan et al., 2009). In this study, the one-step carbonization activation method by freeze-thaw premixed treatment could make KOH and Urea more evenly dispersed into the carbon source to achieve uniform pore size distribution and higher N content, compared to the mechanical physical method.

### 3.3 Gas adsorption and separation performance

**FIGURE 6:**
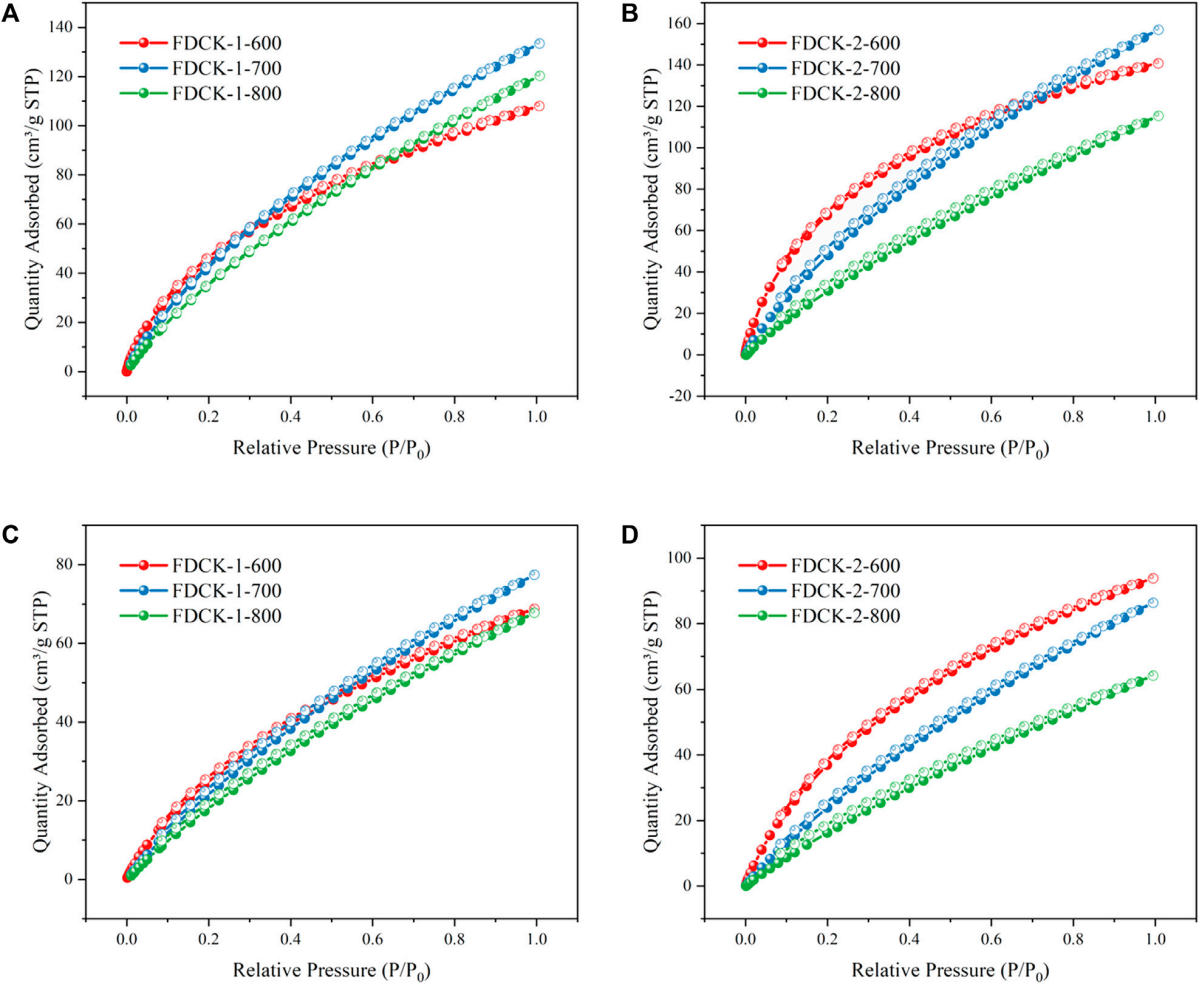
CO_2_ adsorption-desorption isotherms of FDCK-x-t at 273 K **(A, B)** and 298 K **(C, D)**.

Figure 6 showed the CO_2_ adsorption isotherms of FDCK-x-t at 1 bar, 273 K, and 298 K. All adsorption and desorption curves coincide, showing good reversibility. There was no obvious hysteresis curve, indicating that the adsorbed gas could be well removed in the desorption process. The adsorbent was easy to regenerate under a vacuum without consuming excess energy. Supplementary Table S3 summarizes the CO_2_ adsorption capacity of the material under different conditions. The materials FDCK-1-700, FDCK-2-600, and FDCK-2-700 exhibit excellent CO_2_ adsorption performance. At 1 bar and 273 K, the CO_2_ adsorption capacities were as follows: FDCK-1-700 has a performance of 5.92 mmol/g, FDCK-2-600 shows a performance of 6.25 mmol/g, and FDCK-2-700 demonstrates a CO_2_ adsorption capacity of 6.97 mmol/g. At 1 bar and 298 K, the CO_2_ adsorption capacities were as follows: FDCK-1-700 has a performance of 3.11 mmol/g, FDCK-2-600 shows a performance of 3.77 mmol/g, and FDCK-2-700 demonstrates a CO_2_ adsorption capacity of 3.47 mmol/g.

**FIGURE 7:**
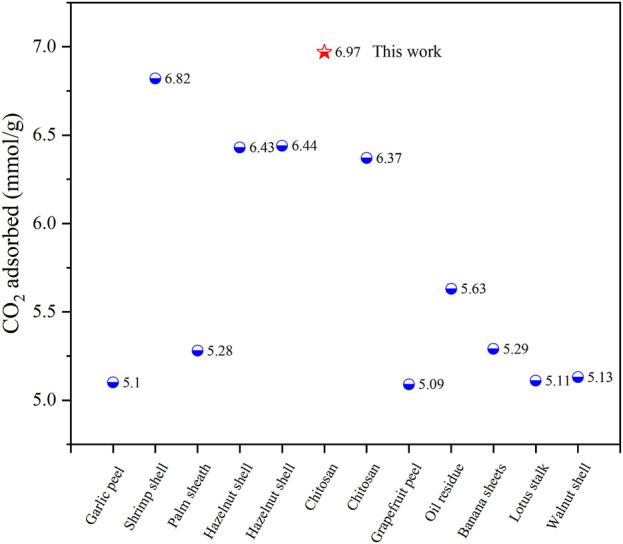
In recent 3 years, the CO_2_ performance of different biomass carbon materials was compared at 1 bar and 273 K.

This CO_2_ adsorption performance was superior to most porous carbon materials reported in recent years, comparison of CO_2_ adsorption performance in this work and recently reported data was shown in Table 3 and Figure 7. It was also superior to some other benchmark adsorbent materials, such as Zeolite 13X (3.5 mmol/g) (Jong-Seok Lee et al., 2002), Ni-4PYC (4.0 mmol/g) (Nandi S et al., 2015), CTF-TPC (4.2 mmol/g) (Dey et al., 2016), NJU-Bai (6.21 mmol/g) (Duan et al., 2012).

**TABLE 3:** Comparison of CO_2_ adsorption performance of biomass-derived porous carbon in recent 3 years.

Precursors	Activator conditions	S_BET_ (m^2^/g)	V_micro_ (cm^3^/g)	CO_2_ uptake (mmol/g) 1bar		References
				273 K	298 K	
Garlic peel	KOH, 700°C	1,248	0.68	5.1	4.1	Huang et al. (2019)
Shrimp shell	KOH, 700°C	1759	0.66	6.82	3.77	Yang et al. (2018)
Palm sheath	KOH, 650°C	840	0.35	5.28	3.48	Zhang et al. (2022)
Hazelnut shell	KOH, 550°C	1,600	0.61	6.43	4.30	Ma et al. (2022)
	KOH, 650°C	1816	0.72	6.44	4.08	Ma et al. (2022)
Chitosan	KOH, 800°C	1,746	1.04	6.37	3.91	Rehman and Park (2020)
Grapefruit peel	KOH, 600°C	2,996	1.33	5.09	-	Li et al. (2022b)
Oil residue	NaNH_2_, 500°C	2,113	0.94	5.63	3.51	Yang et al. (2020a)
Banana sheets	KOH, 800°C	1,988	0.67	5.29	4.16	Li et al. (2020b)
Lotus stalk	KOH, 600°C	1,188	0.43	5.11	3.68	Yang et al. (2020b)
Walnut shell	KOH, 850°C	2,354	0.97	5.13	3.04	Yang et al. (2020c)
Chitosan	KOH, 600°C	1,255	0.575	6.25	3.77	This Work
	KOH, 700°C	2,457	1.12	5.92	3.11	This Work
	KOH, 700°C	2,464	1.151	6.97	3.47	This Work

It was worth noting that at the same KOH/chitosan ratio, FDCK-x-600 showed higher CO_2_ adsorption capacity at low pressure (<0.3 bar), which could be attributed to the high nitrogen content of FDCK-x-600. As shown in Supplementary Table S2, Under 273 K and 0.15 bar conditions, FDCK-2-600 had the maximum CO_2_ adsorption capacity of 2.56 mmol/g. This performance was superior to or comparable to some adsorbent materials, such as PSK-2-650 (2.0 mmol/g) (Zhang et al., 2022), SNMC-2-600 (2.21 mmol/g) (Zhang et al., 2019), NJU-Bai (1.5 mmol/g) (Duan et al., 2012).

**FIGURE 8:**
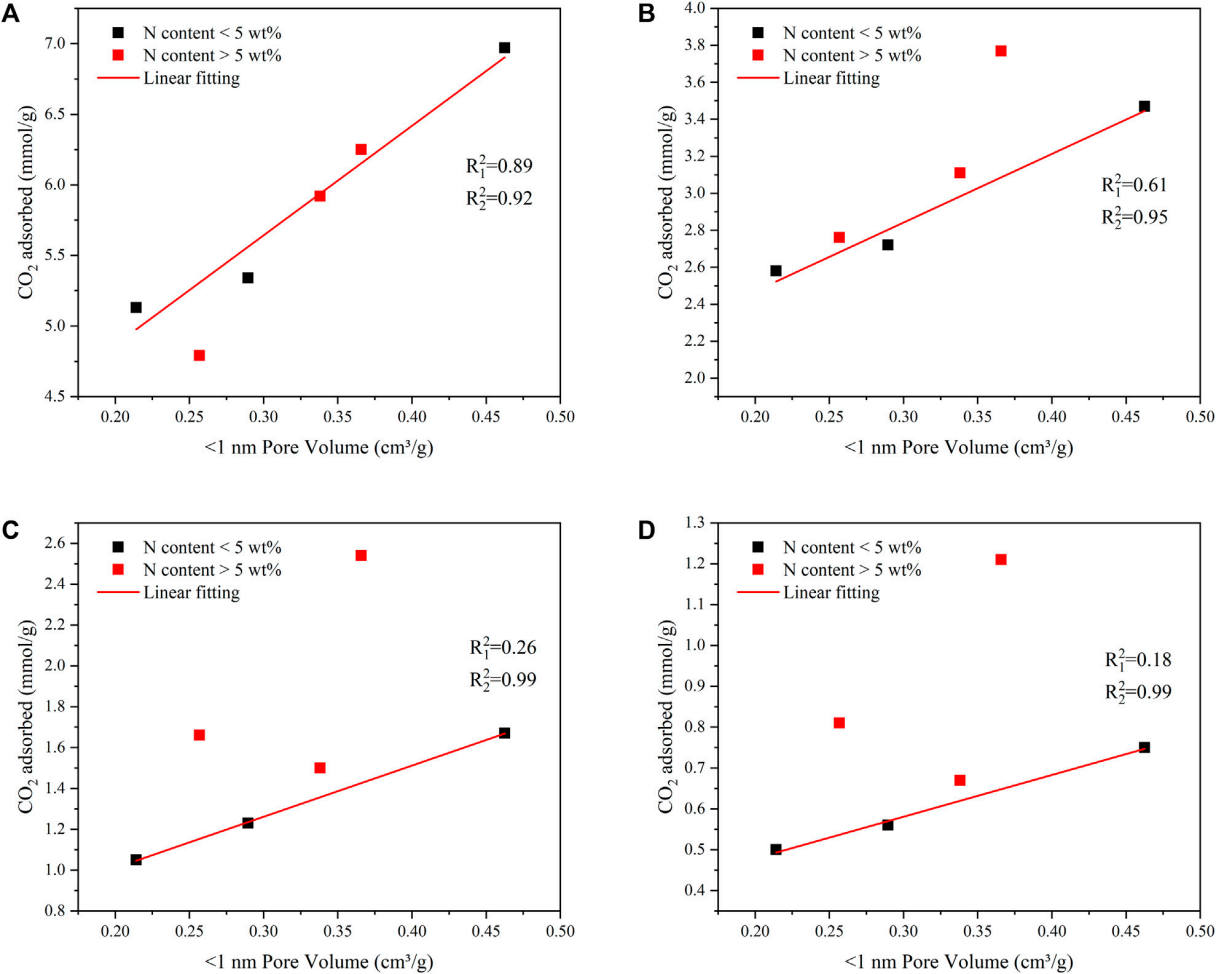
The linear relationship of the narrow pore volume and CO_2_ capture capacity at 1 bar and 273 K **(A)**, 1 bar and 298 K **(B)**, 0.15 bar and 273 K **(C)**, 0.15 bar and 298 K **(D)**.

To further study the effect of extremely narrow micropores and nitrogen content on CO_2_ absorption capacity. Figure 8 showed the regression model of extremely narrow micropore volume and CO_2_ absorption. $R_{1}^{2}$ was the regression coefficient of all points fitting, and $R_{2}^{2}$ was the regression coefficient of fitting after screening out points with N content exceeding 5 wt%. The results showed a high regression coefficient between CO_2_ absorption and very narrow pore volume at the condition of 1bar and 273K. High nitrogen content (nitrogen content >5 wt%) had little effect on the regression model ( $R_{1}^{2}$ = 0.89 VS $R_{2}^{2}$ = 0.92). It could be observed that high nitrogen content significantly improved CO_2_ adsorption performance under the condition of 1bar and 298 K. FDCK-2-600 with a nitrogen content of 6.47 wt% exhibited higher CO_2_ adsorption performance than FDCK-2-700 (nitrogen content of 1.2 wt%) with the maximum extremely narrow pore volume at 298 K at 1bar. At 0.15 bar, high nitrogen content had a significant effect on the regression model ( $R_{1}^{2}$ = 0.26 VS $R_{2}^{2}$ = 0.99 at 273 K; $R_{1}^{2}$ = 0.18 VS $R_{2\;}^{2}$ = 0.99 at 298 K). Among them, FDCK-1-600 with a nitrogen content of 13.08 wt% showed much higher CO_2_ adsorption capacity than FDCK-1-800 (nitrogen content was 4.4 wt%), and the two samples had similar extremely narrow microporous content (0.2729 VS 0.289 cm^3^/g).

The results showed that the adsorption capacity for CO_2_ gas was determined both by the extremely narrow pore volume and nitrogen content. When the pressure was 1 bar, the volume of extremely narrow micropores played a leading role in the CO_2_ adsorption performance of FDCK-x-t. However, under low pressure, the influence of the nitrogen content on the CO_2_ adsorption performance of materials was more significant.

**FIGURE 9:**
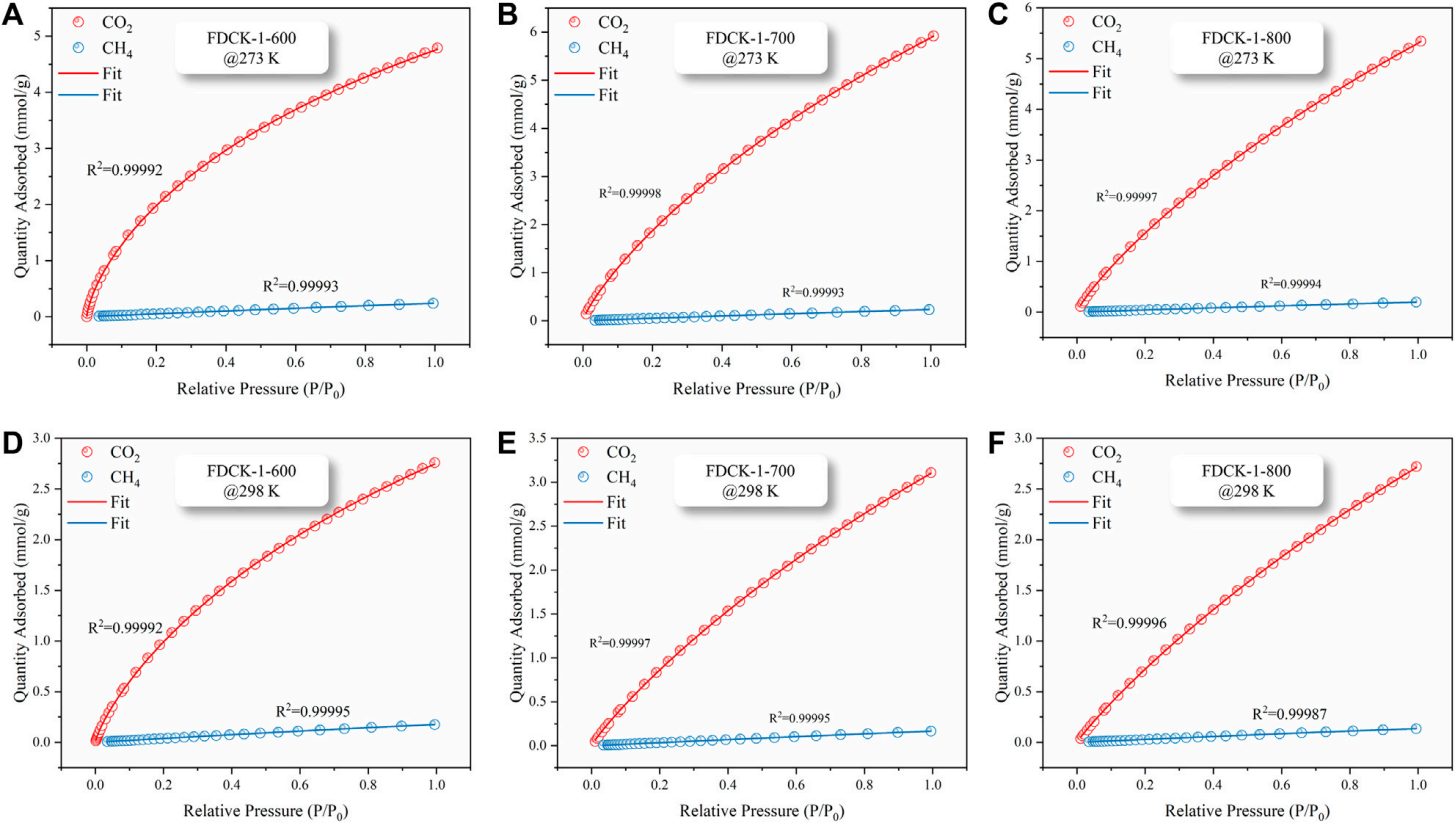
CO_2_ and CH_4_ isothermal adsorption fitting curves, FDCK-1-t at 273 K **(A–C)**, FDCK-1-t at 298 K **(D–F)**.

**FIGURE 10:**
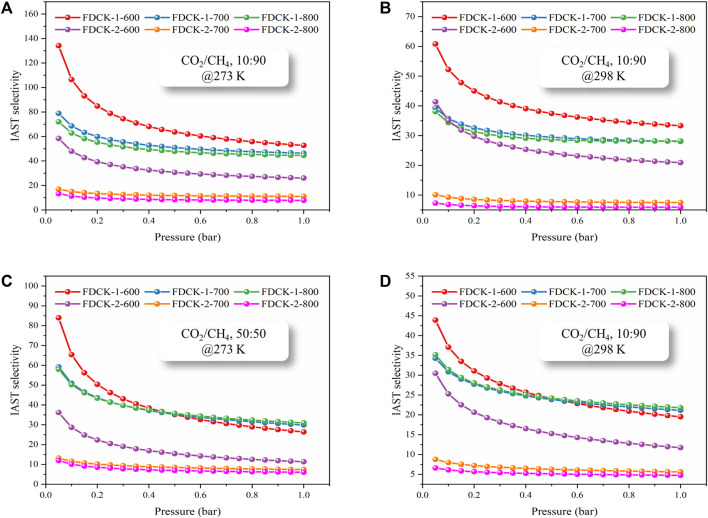
The IAST selectivity of FDCK-x-t at different conditions, CO2/CH4 10:90 and 273 K **(A)**, CO_2_/CH_4_ 10:90 and 298 K **(B)**, CO2/CH4 50:50 and 273 K **(C)**, CO2/CH4 50:50 and 298 K **(D)**.

To obtain the CO_2_/CH_4_ selectivity of FDCK-x-t, its CH_4_ adsorption capacity was measured under 1 bar, 273 K, and 298 K (the adsorption and desorption curves were shown in Supplementary Figure S3, and the adsorption capacity was demonstrated in Supplementary Table S3). The Langmuir-Freundlich model (Li Y. et al., 2020) was used to fit the CO_2_ and CH_4_ adsorption isotherms of FDCK-x-t to evaluate the relative performance of adsorbents through the adsorption isotherms of single-component gas (Figure 9 and Supplementary Figure S4). In consideration of the significant difference in gas concentration ratio (volume ratio) in natural biogas, the selectivity coefficient of CO_2_/CH_4_ at 10: 90 and 50: 50 was calculated in this study. Figure 10 and Table 4 showed and summarized the selectivity results calculated using IAST. It was noteworthy that FDCK-2-700 showed a relatively low IAST selectivity of 11.1 (7.5), although it showed a high CO_2_ adsorption performance of 6.97 mmol/g (3.47 mmol/g) at the conditions of 1 bar, 273 K (298 K) and CO_2_/CH_4_ (10: 90). FDCK-1-600 showed moderate CO_2_ adsorption performance at 1 bar, 273 K (298 K), but showed a surprising IAST selectivity of 52.7 (33.3) at this condition. This was mainly attributed to FDCK-1-600 having the highest nitrogen content (13.08 wt%). At the same time, compared with the preparation of nitrogen-doped porous carbon materials CK-1-700 by physical mixing under the same conditions, the IAST selectivity of FDCK-1-700 was improved by more than two times. The results showed that compared with the mechanical and physical mixing method, the one-step carbonization activation method of freeze-thaw premixed treatment had apparent advantages in gas adsorption and separation.

**TABLE 4:** IAST selectivity of the FDCK-x-t at different conditions and CO_2_ isosteric heat of adsorption.

Specimens	CO_2_/CH_4_ (10: 90)		CO_2_/CH_4_ (50: 50)		Q_st_ (KJ/mol)
	273 K	298 K	273 K	298 K	
CK-1-700	11.7	7.3	7.9	5.2	26.06
FDCK-1-600	52.7	33.3	26.4	19.5	27.35
FDCK-1-700	46.4	28.0	29.9	21.1	24.35
FDCK-1-800	44.5	28.2	30.9	21.8	23.69
FDCK-2-600	26.0	20.9	11.3	11.7	30.51
FDCK-2-700	11.1	7.5	7.4	5.6	25.21
FDCK-2-800	7.8	5.9	6.0	4.8	22.07

The results showed that FDCK-x-t had good selective adsorption and separation performance for CO_2_/CH_4_, which was mainly attributed to the high nitrogen content in FDCK-x-t. The introduction of polar N-containing species in carbon materials enhanced the van der Waals force on CO_2_ intermolecular with quadrupole moment but had little effect on non-polar CH_4_ (Xiang et al., 2012). FDCK-x-t showed excellent IAST selectivity of 33.3 (52.7) and 21.8 (30.9) at 1 bar, 298 K (273 K) at two different gas mixtures (CO_2_/CH_4_, 10: 90; CO_2_/CH_4_, 50: 50). This property was superior to many porous carbon materials and some other reference adsorbent materials, as shown in Table 5.

**TABLE 5:** The selectivity of the prepared porous carbon materials to different ratios of CO_2_/CH_4_ at 1 bar and 273 K was compared with that of reported adsorbent materials.

Specimens	CO_2_/CH_4_ (10: 90)	CO_2_/CH_4_ (50: 50)	
ACSs-N	8.19 (7.49)^a^	\	Li et al. (2020a)
PSK-1-550	7.1	\	Zhang et al. (2022)
APCN-t	4.5 (5.1)^a^	\	Qin et al. (2019)
Silicalite-1	2.6	\	Yang et al. (2013)
OAC-2	\	4.6	Wang et al. (2015)
CC-CH	\	8.6	Jung et al. (2021)
N-WAPC	\	3.03 (3.19)^a^	Li et al. (2019)
SC700P	\	7.0	Mestre et al. (2014)
MOF-505@5GO	\	8.6	Chen et al. (2017)
MKPOP-4	\	4.7	Li et al. (2016)
FDCK-1-600	52.7 (33.3)^a^	26.4 (19.5)^a^	This Work
FDCK-1-800	44.5 (28.2)^a^	30.9 (21.8)^a^	This Work

^a^ The selectivity value at 1 bar and 298 K.

**FIGURE 11:**
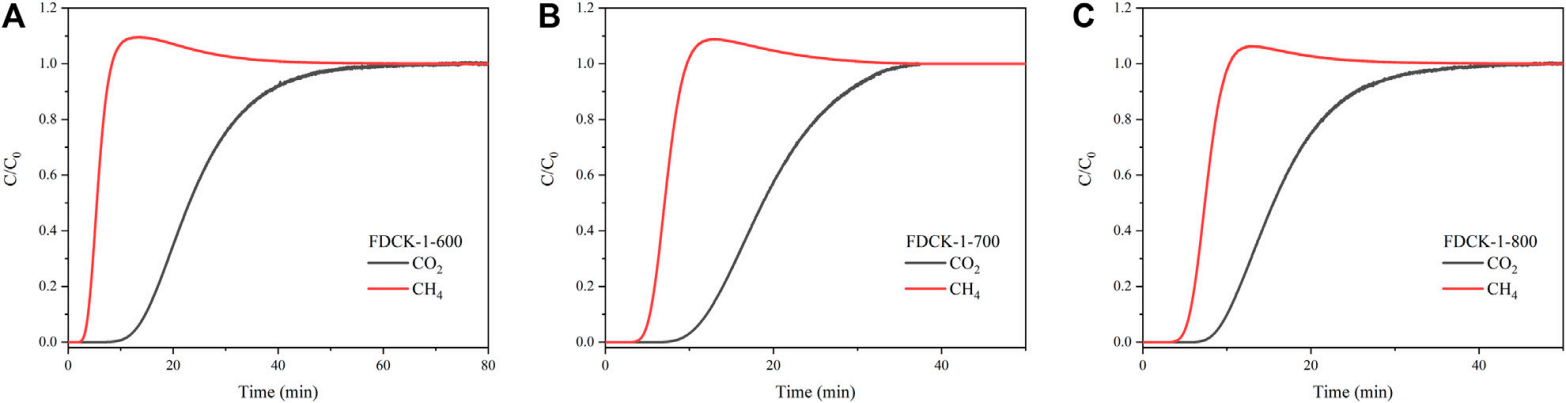
The breakthrough curves of CO_2_/CH_4_ (10:90) at 25°C and 1 bar, FDCK-1-600 **(A)**, FDCK-1-700 **(B)**, FDCK-1-800 **(C)**.

To verify the feasibility of FDCKs in practical application, a dynamic breakthrough experiment was carried out with FDCK-1-t in CO_2_/CH_4_ (10: 90) gas mixture. As shown in Figure 11, CH_4_ was detected earlier when the mixed gas passed through the column filled with adsorbent. For FDCK-1-600, the breakthrough point of CH_4_ was 3.7 min, the breakthrough point of CO_2_ was 13.2 min, and the penetration time was 9.5 min. For FDCK-1-700, the breakthrough point of CH_4_ was 5.2 min, the breakthrough point of CO_2_ was 10.9 min, and the penetration time was 5.7 min. For FDCK-1-800, the breakthrough point of CH_4_ was 5.5 min, the breakthrough point of CO_2_ was 9.4 min, and the penetration time was 3.9 min. It could be seen that FDCKs could selectively adsorb CO_2_ in the actual CO_2_ and CH_4_ mixture gas for biogas upgrading. The results showed that FDCKs had a broad application prospect in biogas selective adsorption.

**FIGURE 12:**
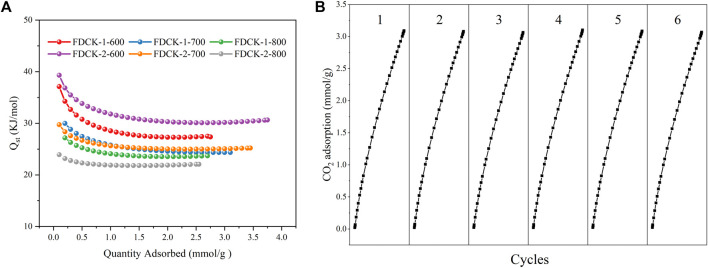
The CO_2_ isosteric heat of adsorption **(A)**, recycling runs of CO_2_ adsorption-desorption on FDCK-1-700 at 1 bar and 298 K **(B)**.

Isosteric heat of adsorption (Q_st_) was a critical thermodynamic parameter to evaluate the interaction between adsorbent and adsorbed gas. According to the CO_2_ adsorption isotherms of adsorbent at 1 bar, 273 K, and 298 K, the relevant Q_st_ values were calculated by the Clausius−Clapeyron equation. The Q_st_ curve of FDCK-x-t was shown in Figure 12A. The initial Q_st_ range of FDCK-x-t at low CO_2_ absorption capacity was 23.9∼39.3 kJ/mol, then gradually decreased to a certain value. It indicated that the surface of these adsorbents had heterogeneous adsorption. FDCK-x-t showed a high initial Q_st_ value and then gradually decreased with the increase of CO_2_ loading, which may be because CO_2_ was first adsorbed in nitrogen-containing functional group adsorption sites and very narrow micropores, and then adsorbed in larger diameter micropores. The higher the Q_st_, the stronger the affinity of the adsorbent for CO_2_, which was conducive to the removal of CO_2_ from biogas. The overall Q_st_ value varies from 21.2 to 39.3 kJ/mol, reflecting the physical adsorption characteristics of the adsorption process, indicating that the material was easy to regenerate.

The regeneration performance was the key to the practical application of carbon dioxide adsorbents. Renewable and reused adsorbents not only save costs but also reduce the recovery and treatment of waste adsorbents. As shown in Figure 12B, six-time CO_2_ adsorption-desorption cycles were performed at 25°C and 1 bar on FDCK-1-700. In the cycle test, the adsorbent was degassed at 25°C for 1 h and then reused for adsorption measurement. The results showed that the adsorption capacity of FDCK-1-700 had little change after 6 times of continuous operation, indicating that the material had an excellent renewable performance.

## 4 Conclusion

In summary, we have successfully developed a one-step carbonization activation method with freeze-thaw pre-mix treatment to prepare extremely narrow microporous nitrogen-doped carbon materials, referred to as FDCKs. These FDCK materials exhibit outstanding performance in both CO_2_ adsorption and gas selective adsorption. They feature controllable nitrogen content, a high specific surface area, a large pore volume, and high CO_2_ adsorption capacity (6.97 mmol/g and 3.77 mmol/g at 1 bar, 273 K, and 298 K, respectively). Moreover, they demonstrate high selectivity at different CO_2_/CH_4_ ratios, offering significant potential for gas mixture separation. These materials maintain stability over multiple adsorption and desorption cycles, ensuring consistent performance through repeated use. In conclusion, FDCK materials hold great promise for various applications, particularly in gas separation and efficient purification, contributing to the reduction of greenhouse gas emissions and the achievement of more environmentally sustainable energy production goals.

## Data Availability

The original contributions presented in the study are included in the article/Supplementary Material, further inquiries can be directed to the corresponding authors.
