# Supplementary material for: N-rich chitosan-derived porous carbon materials for efficient CO2 adsorption and gas separation
Source: Front Chem. 2023 Dec 14;11:1333475. doi: 10.3389/fchem.2023.1333475 (PMC10752987; doi:10.3389/fchem.2023.1333475)
Supplement: Supplementary file 1 [file DataSheet1.docx]

**N-rich chitosan-derived porous carbon materials for efficient CO_2_ adsorption and gas separation**


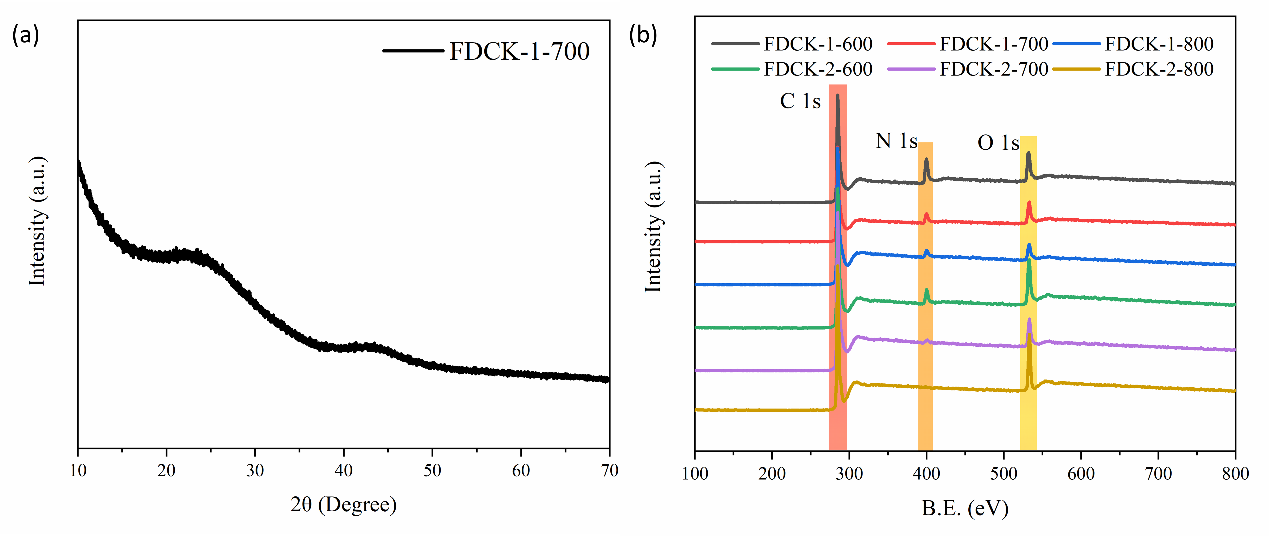


Fig. S1 (a) XRD pattern of FDCK-1-700 (b) XPS patterns of FDCK-x-t





Fig. S2 (a) C 1s XPS spectra (b) N 1s XPS spectra (c) O 1s XPS spectra

Table S1 The relative content of different N functional groups

| Specimens | Pyridinic N (%) | Pyrrolic N (%) | Quaternary N (%) |
| --- | --- | --- | --- |
| FDCK-1-600 | 35 | 45 | 21 |
| FDCK-1-700 | 35 | 41 | 24 |
| FDCK-1-800 | 32 | 39 | 29 |
| FDCK-2-600 | 24 | 52 | 24 |


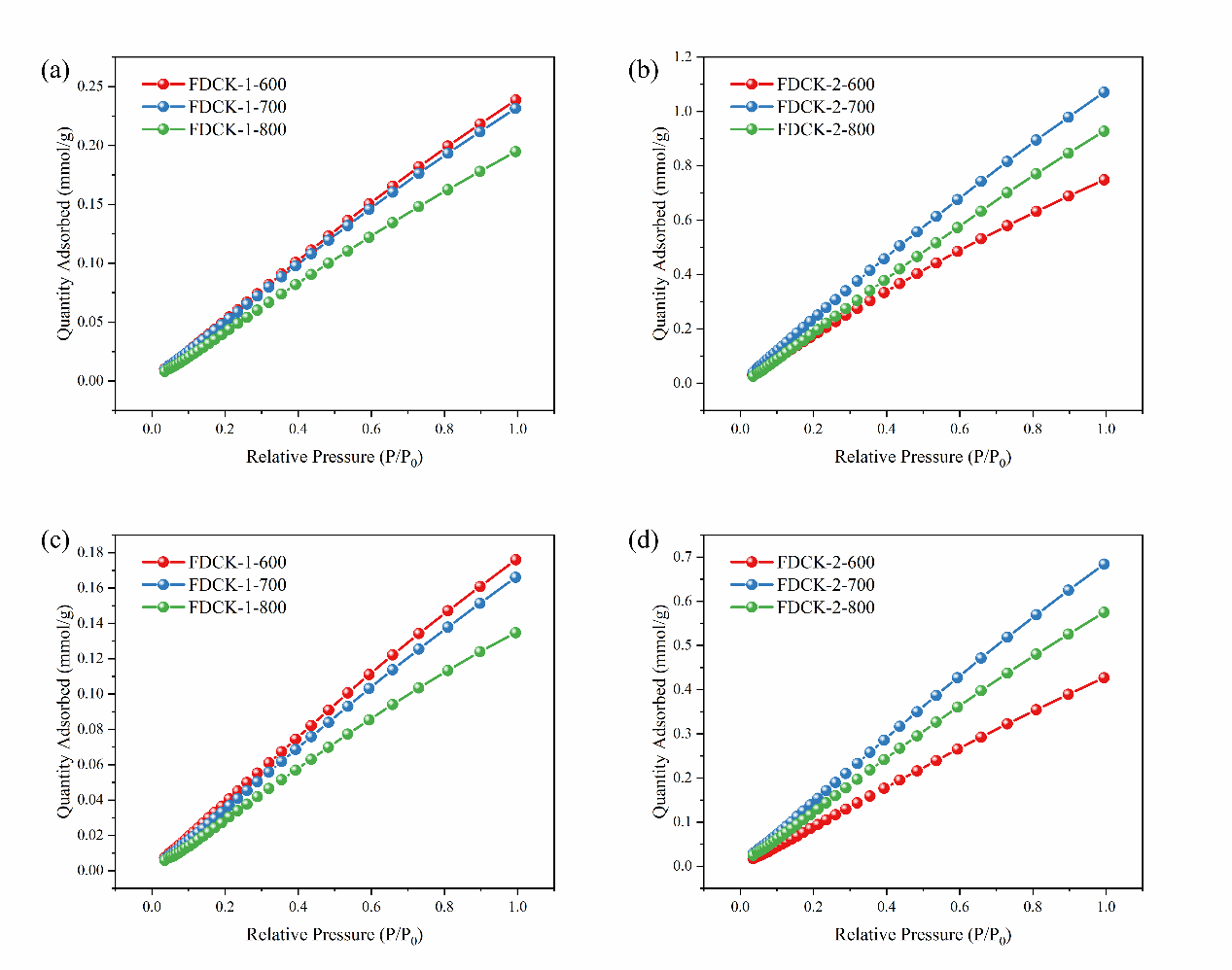


Fig. S3 CH_4_ adsorption isotherms of FDCK-x-t at (a, b) 273 K and (c, d) 298 K


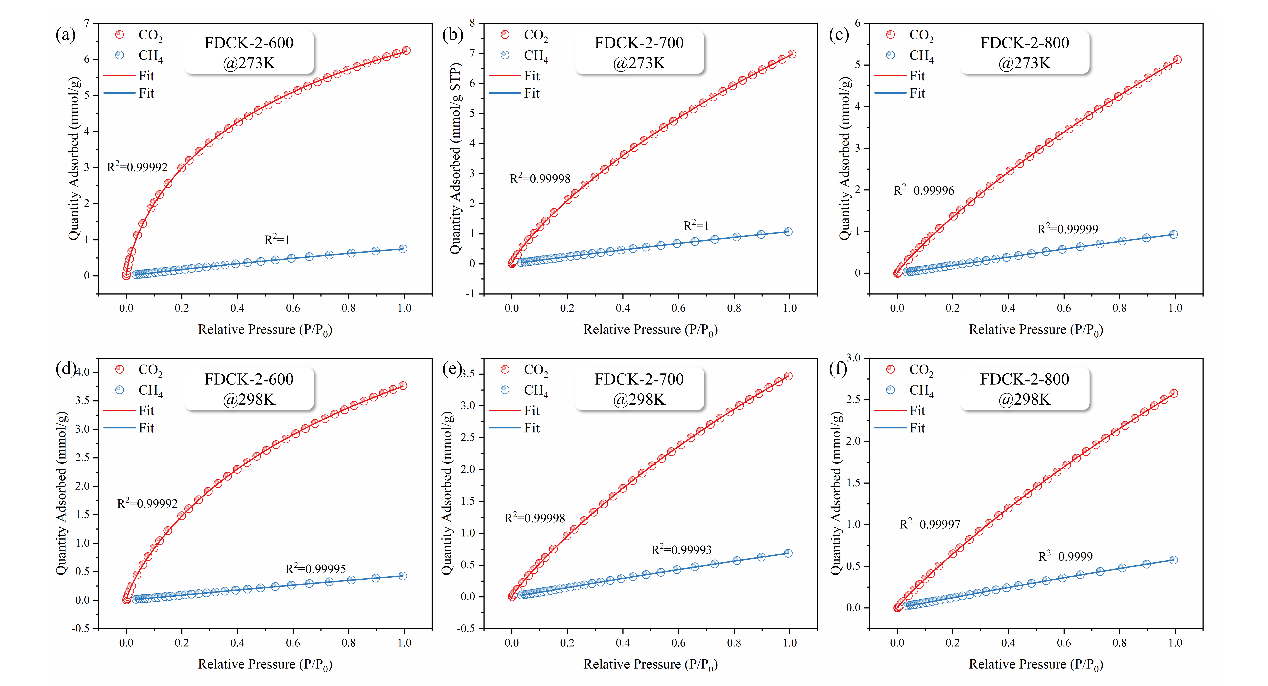


Fig. S4 CO_2_ and CH_4_ isothermal adsorption fitting curve

Table S2 Adsorption paramatres of CO_2_ and CH_4_ at 0.15 bar

| Specimens | CO_2_ (mmol/g) | |  |
| --- | --- | --- | --- |
|  | 273 K | 298 K |  |
| CK-1-700 | 1.26 | 0.54 |  |
| FDCK-1-600 | 1.66 | 0.81 |  |
| FDCK-1-700 | 1.50 | 0.67 |  |
| FDCK-1-800 | 1.23 | 0.56 |  |
| FDCK-2-600 | 2.54 | 1.21 |  |
| FDCK-2-700 | 1.67 | 0.75 |  |
| FDCK-2-800 | 1.05 | 0.50 |  |


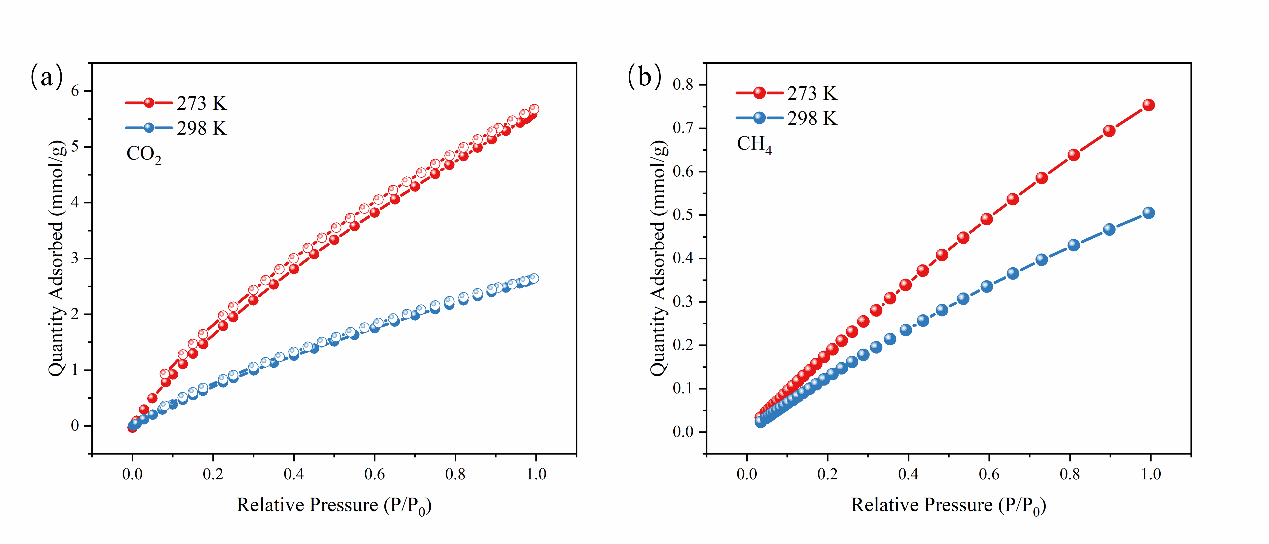
Fig. S5 CO_2_ and CH_4_ adsorption isotherms of CK-1-700


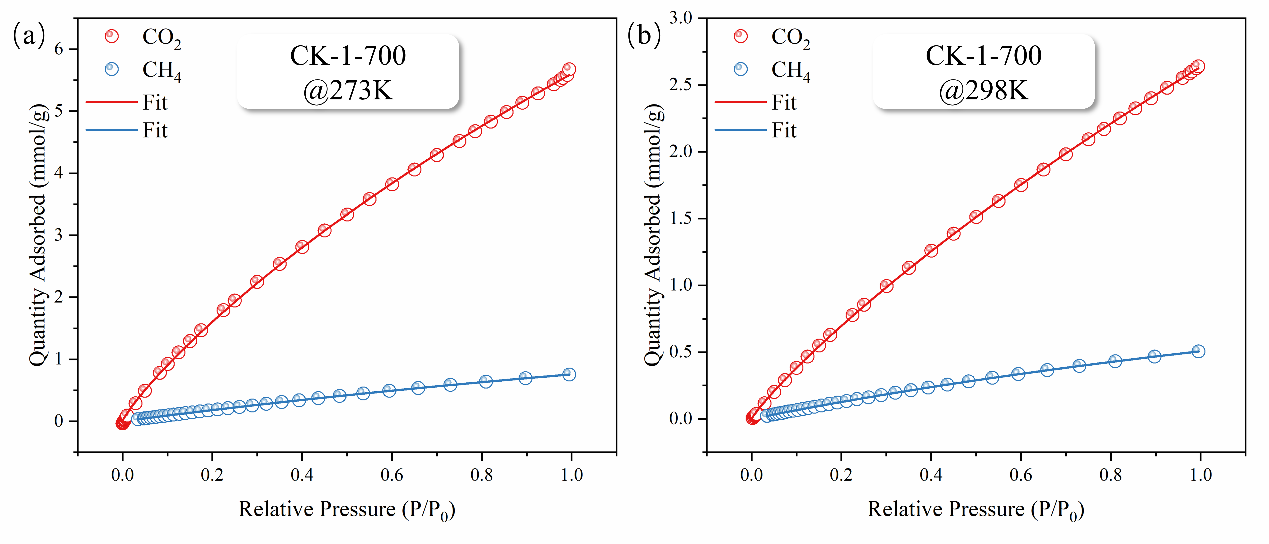


Fig. S6 CO_2_ and CH_4_ isothermal adsorption fitting curve

Table S3 Adsorption paramatres of CO_2_ and CH_4_

| Specimens | CO_2_ (mmol/g) | |  | CH_4_ (mmol/g) | |
| --- | --- | --- | --- | --- | --- |
|  | 273 K | 298 K |  | 273 K | 298 K |
| CK-1-700 | 5.67 | 2.64 |  | 0.75 | 0.50 |
| FDCK-1-600 | 4.79 | 2.76 |  | 0.24 | 0.18 |
| FDCK-1-700 | 5.92 | 3.11 |  | 0.23 | 0.17 |
| FDCK-1-800 | 5.34 | 2.72 |  | 0.19 | 0.13 |
| FDCK-2-600 | 6.25 | 3.77 |  | 0.75 | 0.43 |
| FDCK-2-700 | 6.97 | 3.47 |  | 1.07 | 0.68 |
| FDCK-2-800 | 5.13 | 2.58 |  | 0.93 | 0.57 |


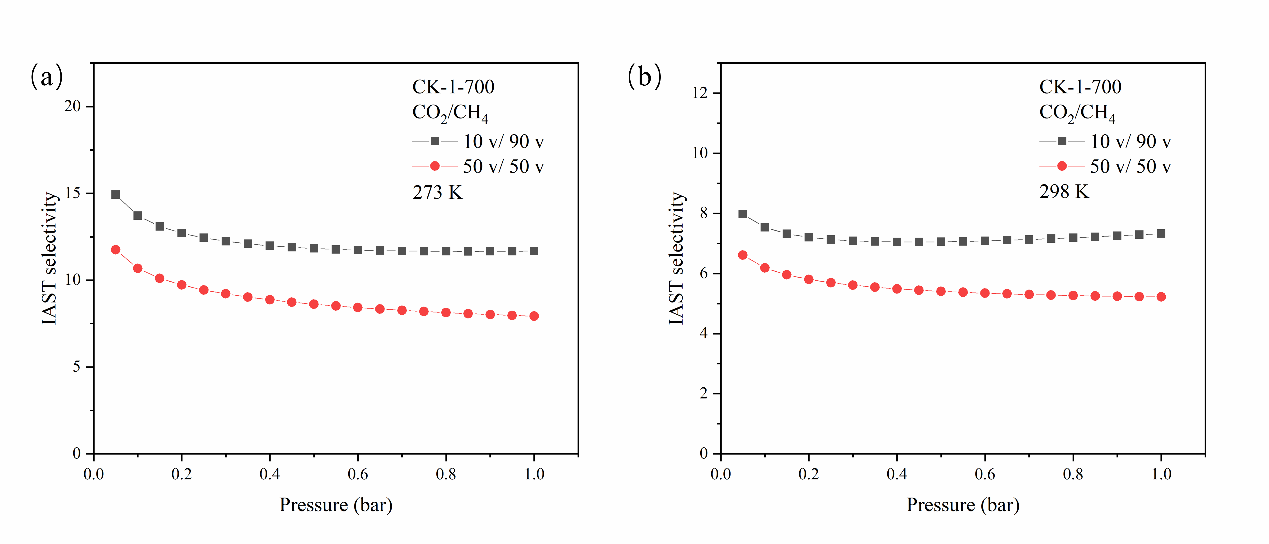


Fig. S7 The IAST selectivity of CK-1-700 at different conditions





Fig. S8 The CO_2_ isosteric heat of adsorption
